# Brain Maturation, Cognition and Voice Pattern in a Gender Dysphoria Case under Pubertal Suppression

**DOI:** 10.3389/fnhum.2017.00528

**Published:** 2017-11-14

**Authors:** Maiko A. Schneider, Poli M. Spritzer, Bianca Machado Borba Soll, Anna M. V. Fontanari, Marina Carneiro, Fernanda Tovar-Moll, Angelo B. Costa, Dhiordan C. da Silva, Karine Schwarz, Maurício Anes, Silza Tramontina, Maria I. R. Lobato

**Affiliations:** ^1^Gender Identity Program, Hospital de Clinicas de Porto Alegre, Universidade Federal do Rio Grande do Sul, Porto Alegre, Brazil; ^2^Department of Physiology, Universidade Federal do Rio Grande do Sul, Porto Alegre, Brazil; ^3^Service of Endocrinology, Hospital de Clínicas de Porto Alegre, Porto Alegre, Brazil; ^4^Instituto D'Or de Pesquisa e Ensino, Rio de Janeiro, Porto Alegre, Brazil; ^5^Institute for Biomedical Sciences, Federal University of Rio de Janeiro, Rio de Janeiro, Brazil; ^6^Graduate Program in Psychology, Pontifícia Universidade Católica do Rio Grande do Sul, Porto Alegre, Brazil; ^7^Division of Medicine Physics, Hospital de Clínicas de Porto Alegre, Porto Alegre, Brazil; ^8^Child and Adolescent Psychiatry Service, Hospital de Clínicas de Porto Alegre, Porto Alegre, Brazil; ^9^Psychiatry and Forensic Medicine Service, Hospital de Clínicas de Porto Alegre, Porto Alegre, Brazil

**Keywords:** gender dysphoria, pubertal suppression, white matter, WISC-IV, cognition

## Abstract

**Introduction:** Gender dysphoria (GD) (DMS-5) is a condition marked by increasing psychological suffering that accompanies the incongruence between one's experienced or expressed gender and one's assigned gender. Manifestation of GD can be seen early on during childhood and adolescence. During this period, the development of undesirable sexual characteristics marks an acute suffering of being opposite to the sex of birth. Pubertal suppression with gonadotropin releasing hormone analogs (GnRHa) has been proposed for these individuals as a reversible treatment for postponing the pubertal development and attenuating psychological suffering. Recently, increased interest has been observed on the impact of this treatment on brain maturation, cognition and psychological performance.

**Objectives:** The aim of this clinical report is to review the effects of puberty suppression on the brain white matter (WM) during adolescence. WM Fractional anisotropy, voice and cognitive functions were assessed before and during the treatment. MRI scans were acquired before, and after 22 and 28 months of hormonal suppression.

**Methods:** We performed a longitudinal evaluation of a pubertal transgender girl undergoing hormonal treatment with GnRH analog. Three longitudinal magnetic resonance imaging (MRI) scans were performed for diffusion tensor imaging (DTI), regarding Fractional Anisotropy (FA) for regions of interest analysis. In parallel, voice samples for acoustic analysis as well as executive functioning with the Wechsler Intelligence Scale (WISC-IV) were performed.

**Results:** During the follow-up, white matter fractional anisotropy did not increase, compared to normal male puberty effects on the brain. After 22 months of pubertal suppression, operational memory dropped 9 points and remained stable after 28 months of follow-up. The fundamental frequency of voice varied during the first year; however, it remained in the female range.

**Conclusion:** Brain white matter fractional anisotropy remained unchanged in the GD girl during pubertal suppression with GnRHa for 28 months, which may be related to the reduced serum testosterone levels and/or to the patient's baseline low average cognitive performance.Global performance on the Weschler scale was slightly lower during pubertal suppression compared to baseline, predominantly due to a reduction in operational memory. Either a baseline of low average cognition or the hormonal status could play a role in cognitive performance during pubertal suppression. The voice pattern during the follow-up seemed to reflect testosterone levels under suppression by GnRHa treatment.

## Introduction

Gender dysphoria (GD), according to DMS-5 (APA-2013), is a condition marked by increasing psychological suffering that accompanies the incongruence between one's experienced or expressed gender and one's assigned gender. Diagnostic criteria consider the individual's developmental history and fit it for two different diagnoses: childhood (pre-pubertal) and adolescence or adulthood, the last share the same DSM-5 (APA-2013) diagnostic criteria. Clinical management is focused on therapy for attenuating dysphoric feelings about the body, as well as body incongruence. These interventions range from biopsychosocial approaches to hormonal treatment and sex-reassignment surgery in adulthood (Coleman et al., 2012).

Childhood and adolescence is a critical time for the development of mental disorders. In this period, GD youths are at high risk of having a clinical diagnosis of depression (Resiner et al., 2015), suicide, self-harm and eating disorders (Connolly et al., 2016; Feder et al., 2017), which are strongly related to dysphoric feelings and to different levels of transphobia exposition (Acerlus et al., 2016).

Preventing the development of secondary sex characteristics is a crucial part of the treatment for alleviating gender inconformity in adolescence (Cohen-Kettenis et al., 2011; Coleman et al., 2012). It is considered a transient and reversible intervention (Smith et al., 2014) and should be prescribed to GD individuals in the Tanner 2–3 stages of puberty development under parents' consent (Tanner, 1962; Hembree et al., 2009; Coleman et al., 2012). Recently, increased interest has been observed on the impact of pubertal suppression on brain maturation, cognition and psychological performance (Staphorsius et al., 2015; Hough et al., 2017, 2019). In our study, we aim to evaluate the WM fractional anisotropy and the IQ scale in a longitudinal case during pubertal suppression.

### Case presentation

An 11-year-old individual, designated a boy at birth, was referred to the Gender Identity Program (PROTIG). The patient fit the criteria for male-to-female (MtF) GD (DSM-5) and gender incongruence (ICD-11 inventory) (Robles et al., 2016), which was translated and adapted for the Brazilian population (Soll et al., 2017). At admission, current psychosis, mood disorders, anxiety and global development disorders were excluded.

The patient was born at term, of normal weight, displayed a male phenotype, and experienced no intercurrences during pregnancy. The parents confirmed normal neuropsychomotor development for each of the developmental milestones. At age three, they noticed some female behaviors and sought out psychological treatment. They were informed about a possible DG diagnosis, and the child was kept in psychotherapy to “reverse the desire of belonging to the opposite sex” until age seven. The patient and parents report that she made efforts to behave as a boy during this treatment. At nine, she assumed her gender identity and reported believing that she was born the wrong sex, and she wanted to be a girl.

At 11 years and 11 months old, she weighed 35.5 kg, was 145.5 cm in height and was in Tanner stage 2, according to male characteristics. The patient's bone age was compatible with the chronological age for both male and female standards. The biochemical and hormonal laboratory tests were normal for age, born-sex, and Tanner stage (testosterone 182 ng/dl, LH 3.3 and FSH 2.2 IU/L). After written consent of the parents and patient for using GnRHa and for publication of data to scientific article, she started receiving Leuprorelin 3.75 mg IM/every 28 days. In the next months, the GnRHa doses were adjusted according to the clinical signs and hormone levels (last testosterone under GnRHa 29 ng/dl). The affective and social domains improved during the GnRH treatment; however, the teachers and school counselors reported some difficulties, specifically in math and exact sciences.

This study was carried out in accordance with the recommendations of the Endocrine Society Clinical Practice Guideline for the Endocrine Treatment of Transsexual Persons (2009, updated in 2017), the Standards of Care for the health of transsexual, transgender and gender-nonconforming people, of the WPATH, (World Professional Association for Transgender Health), 7th ed, 2012, and the local Research Ethics Committee from Hospital de Clinicas de Porto Alegre, with written informed consent from all subjects and their caretakers. All subjects gave written informed consent in accordance with the Declaration of Helsinki. The protocol was approved by the local Research Ethics Committee from Hospital de Clínicas de Porto Alegre.

### Physical exams and laboratory results

Before treatment and during different periods of follow-up, in addition to endocrinological monitoring, specific assessments were performed to evaluate the neuropsychiatric status during GnRHa administration.

### Psychological evaluation

A standard psychological protocol and psychiatric evaluations were applied for personality traits, parental style, life-long depressive symptomatology, and overall intellectual performance assessment at study admission. House-Tree-Person (HTP) (Buck, 2003); Kiddie-Sads-Present and Lifetime (K-SADS-PL) Brazilian version 1.0 (Brasil and Bordin, 1996); Parental Styles Inventory (PIS) (Gomide, 2006); Wechsler Intelligence Scale for Childhood (Brazilian validated version) (WISC-IV) (Rueda et al., 2013) were the chosen instruments.

The K-SADS-PL matched only for major depressive disorder at five, which was the age of the “conversion therapy.” The HTP was normal for age, signaling the desire to belong to the opposite sex (female), and the PIS was at percentile 95, which is the optimal parental style. During the clinical follow-up, that included weekly appointments with a psychotherapist, the patient did not match clinical criteria for psychiatric comorbidities.

Three evaluations were performed for the WISC-IV: T0, prior to GnRHa treatment, T1, 22 months and T2, 28 months after pubertal suppression. The same version of the test was applied by the same professional, and they were performed in the same environment at all set points.

### Longitudinal cognitive WISC-IV evaluation

Comparing the periods of follow-up, a reduction on Global IQ (GIQ) during pubertal suppression was observed. In T1, the GIQ was lower than before hormonal treatment (T0), and this finding was sustained by the third WISC-IV evaluation (Table 1).

**Table 1:** The results of the longitudinal evaluation of the Weschler Scale of Intelligence.

	**Composite scaled score**	**Percentile rank**	**Confidence interval 95%**	**Qualitative description**
**T0-Admission (11 years and 10 months old)**				
Global IQ (GIQ)	80	9	76–86	Low Average
Comprehension Index (VCI)	101	53	94–108	Average
Perceptual Reasoning Index (PRI)	79	8	73–88	Borderline
Working Memory Index (WMI)	83	13	77–91	Low Average
Processing Speed Index (PSI)	68	2	63–81	Extremely Low
**T1- (13 Years and 3 Months Old)**				
Global IQ (GIQ)	71	3	67–77	Borderline
Comprehension Index (VCI)	91	27	84–99	Average
Perceptual Reasoning Index (PRI)	73	4	68–82	Borderline
Working Memory Index (WMI)	68	2	63–77	Extremely Low
Processing Speed Index (PSI)	74	4	68–86	Borderline
**T2- (14 Years and 2 Months Old)**				
Global IQ (GIQ)	70	2	66–76	Borderline
Comprehension Index (VCI)	86	18	80–94	Low Average
Perceptual Reasoning Index (PRI)	77	6	71–86	Borderline
Working Memory Index (WMI)	74	4	69–83	Borderline
Processing Speed Index (PSI)	64	1	59–77	Extremely Low

*A Global IQ reduction is observed. At the end of 28 months of treatment, speed processing and memory remain lower than before GnRHa treatment*.

According to the results obtained through the cognitive evaluations, the patient presented a decrease in their overall intellectual performance after the onset of pubertal block, pointing to immaturity in her cognitive development (Table 1).

### Neuroimaging

A DTI protocol was used to assess the brain's white matter FA, which is an indirect evaluation of neuronal fascicule integrity and maturation. The WM microstructure maturation during puberty was previously reported (Menzies et al., 2015; Pangelinan et al., 2016). The Magnetic Resonance Imaging (MRI) scans were done on a Philips Achieva 1.5T (Bethesda/Netherlands, 2009) with a dedicated 8 channels head coil. The diffusion weighted MRI images were acquired using a single-shot spin-echo echo-planar imaging (SE-EPI) sequence: TR/TE/Flip, angle/Pixel Band with (11,500 ms/80 ms/90/1784 Hz); b-value of 0 and 800 s/mm2 with 32 directions; voxel sizes: 2 × 2 × 2 mm^3^ (high resolution); matrix sizes 112 × 112 × 70, no gaps between slices; and Field of View between 224 × 224 mm. The data were collected at T0, T1, and T2.

The images weighted by the diffusion tensor were initially inspected for the identification of possible artifacts generated during acquisition. After the quality control, the diffusion images and non-weighted diffusion images were co-registered for the correction of movement and distortions caused by eddy currents. From the co-registered images, non-cerebral tissue was removed with the brain extraction tool (FMRIB's Software Library, FSL version 5.0). The six diffusion tensor elements (3 auto-vectors: v1, v2 and v3 and 3 auto-values λ1, λ2 e λ3) and consequent fractional anisotropy (FA) maps were calculated using the DTIFIT, adjusting the data for a tensor diffusion model for each voxel. The colored FA map was used for visualization of the tensor orientation, respecting the white matter tract anatomy.

**Figure 1:**
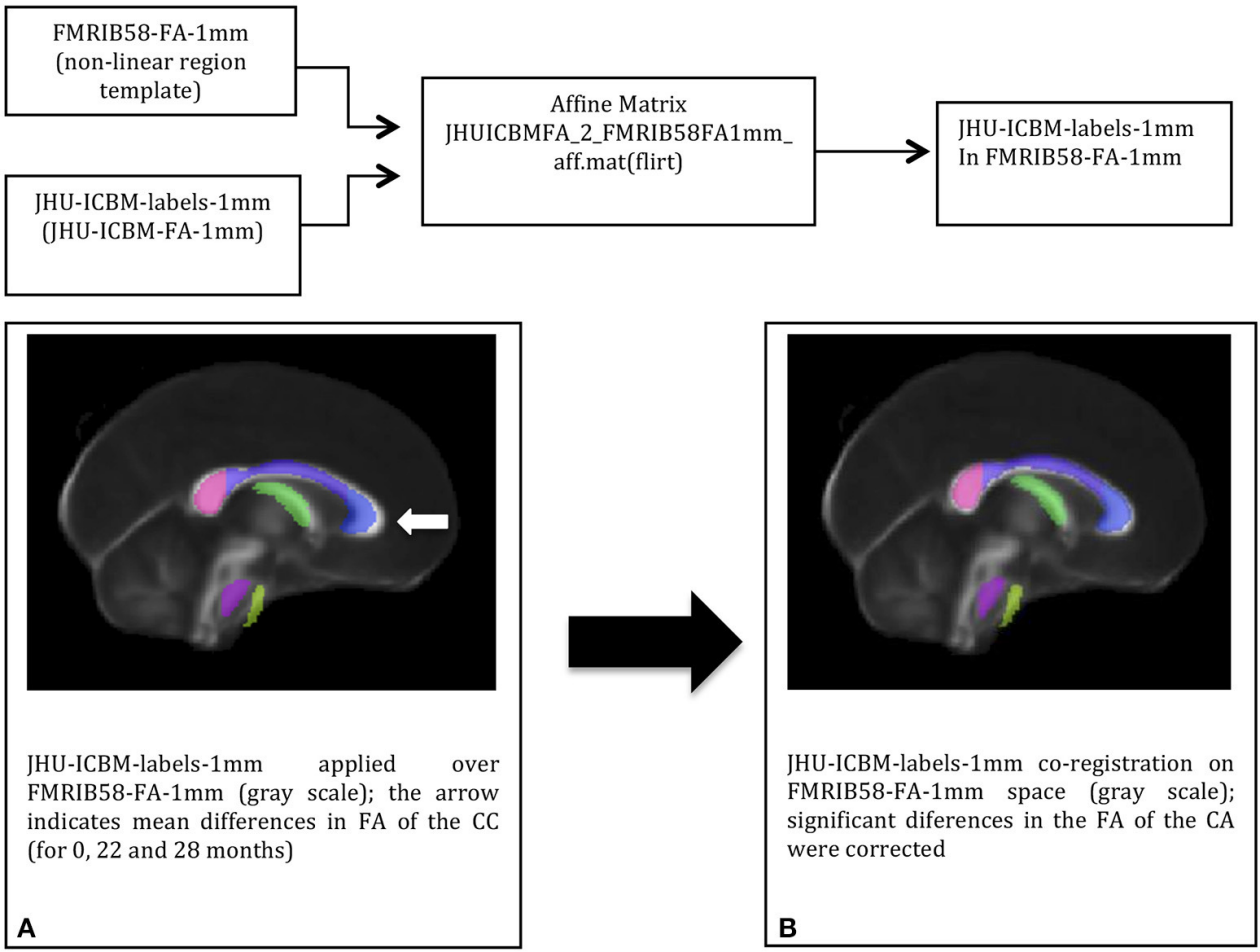
The DTI-based white matter atlas JHU-ICBM-labels-1 mm (colored in transparency) before **(A)** and after **(B)** affine-co-registration with the FSL FA template (in gray scale): co-registration corrected the misalignments (white arrow) between the two images before the atlas transformation to the individual space.

To extract the mean FA value of the genu of the corpus callosum (CC), the white matter (WM) John Hopkins University Atlas JHU-ICBM-labels-1 mm was co-registered with each individual FA map (from T0, T1, and T2). The JHU-FA image, which the JHU-ICBM-labels-1 mm is aligned with, and the FSL FA template were co-registered using an affine transformation to reduce any misalignment between the chosen atlas and the FSL FA template. The JHU atlas was co-registered to this template by applying the resulted affine-registration matrix to the JHU-ICBM-labels-1 mm (Figure 1). After non-linearly registering the FA template to each individual FA map, the genu of the CC from the template-aligned JHU-ICBM-labels-1 mm could also be non-linearly transformed to each individual FA space. Finally, the mean FA from this region of interest (ROI) at each time point could be extracted and compared.

**Figure 2:**
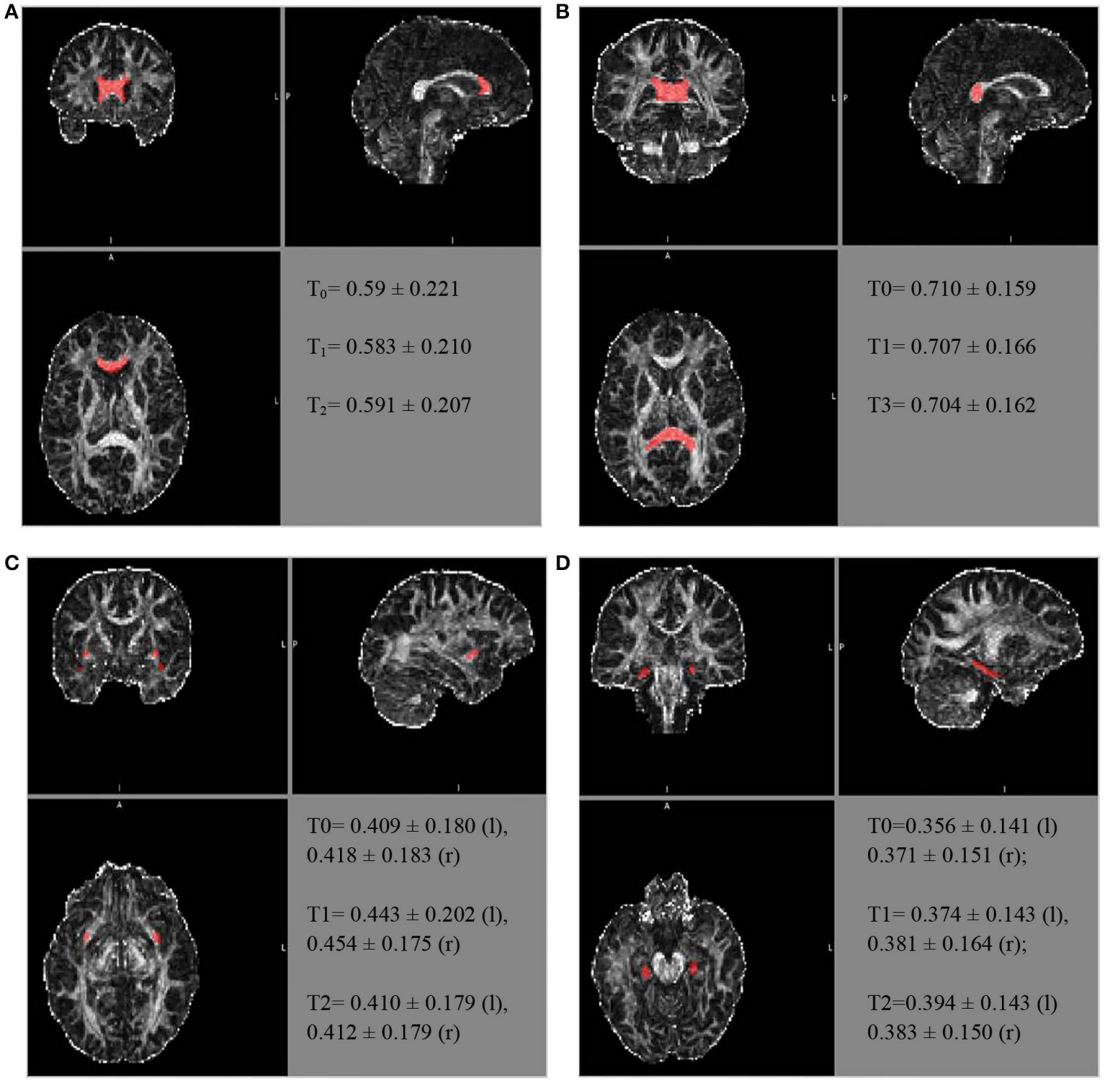
The longitudinal FA variation for different regions of interest. The atlas showing the locations for the **(A)** genu of the CC, **(B)** splenium of the CC, **(C)** uncinate fascicule right r/l, and **(D)** cingulate hippocampal fascicule r/l.

Similarly, further mean FA estimates were performed for the hippocampal cingulate fascicule and the splenium of the CC, bilateral, using the JHU-ICBM-labels-1 mm. The FA estimates were also performed for the bilateral uncinate fascicule from the WM atlas JHU-ICBM-tracts-prob-1 mm, which has better fascicule representation, following the steps described above. Figure 2 presents similar FA values for the different ROIs during the follow-up. The mean FA values of the CC's splenium was 0.710 ± 0.159 before treatment, 0.707 ± 0.166 for T1, and 0.704 ± 0.162 for T2. The mean FA values for the hippocampal cingulate fascicule for T0, T1, and T2 were: 0.356 ± 0.141 (l:left) 0.371 ± 0.151 (r:right); 0.374 ± 0.143 (l), 0.381 ± 0.164 (r); and 0.394 ± 0.143 (l) 0.383 ± 0.150 (r), respectively. For the uncinate fascicule, the results of mean FA values for T0, T1, and T2 were: 0.409 ± 0.180 (l), 0.418 ± 0.183 (r); 0.443 ± 0.202 (l), 0.454 ± 0.175 (r); and 0.410 ± 0.179 (l), 0.412±0.179 (r), respectively.

The FA of the genu of the CC, using the JHU tract probe in MATLAB, presented small variations between T0, T1, and T2 (T_0_ = 0.523473; T_1_ = 0.507267; T_3_ = 0.491564), toward FA reduction. After adjusting by the JHU tract probe FSL, the analyses were similar among the periods of assessment (T_0_ = 0.504 ± 0.216; T_1_ = 0.493 ± 0.205; T_2_ = 0.502 ± 0.2066), as well as after the JHU/Labels, 1 mm (T_0_ = 0.59 ± 0.221; T_1_ = 0.583 ± 0.210; T_2_ = 0.591 ± 0.207), without a reduction trend (Figure 2).

### Voice evaluation

The voice collections were performed at four time points (before treatment and the eighth, seventeenth, and twentieth month of treatment) by means of a sustained vowel/a/. The voice was recorded directly on the computer. The microphone was positioned at an angle of 90° from the mouth, and the same distance of 4 cm between the microphone and mouth was maintained to avoid signal interference. The patient was instructed to emit the vowel sound in a usual tone and intensity. For the acoustic analysis, the first 5 s of the vowel/a/ were used, excluding the beginning of the broadcast so that the vocal attack did not interfere with the data analysis.

The measurements were obtained through the Multidimensional Voice Program Advanced software (MVDPA), Kay Pentax®. We considered the following measures: the mean fundamental frequency (f0) that corresponds to the velocity of the vocal fold vibration (number of glottal cycles per second), the maximum fundamental frequency (FHI), and the minimum fundamental frequency (flo). This measure is directly related to the length, tension, rigidity and mass of the vocal folds during their interaction with the subglottic pressure, which reflects the biomechanical characteristics of the vocal folds. Normal values vary according to sex, age, and physical and laryngeal structures. In Brazil, the reference values are: 80–150 Hz for men, 150–250 Hz for women and above 250 Hz for children (Campisi et al., 2002).

Other measures used were those of frequency disturbance *(jitter)*. The measure of jitter is the fundamental frequency variation in consecutive cycles that reflects the irregularity of the mucosa vibration of the vocal folds. There are different ways to extract these measures, such as those used in this study: Jitter percentage (Jitt), average relative frequency of disturbance (RFD), and pitch perturbation quotient (PPQ). The intensity or shimmer disturbance corresponds to the amplitude variation in consecutive cycles that is present to a certain degree in all vocal samples.

The period of collection of the vocal samples and the results of the acoustic measurements of the voice are shown in Table 2. The voice f0 varied during the first 17 months of treatment, with a decrease of approximately 30 Hz. Then, in the last assessment there was an increase in the f0. The shimmer was not modified. The other measures (jitter and PPQ) also confirmed the variability in the fundamental frequency that occurred during the evaluation period.

**Table 2:** The longitudinal vocal acoustic measures according to the period of collection of the vocal sample.

**Voice acoustic measures**	**Time 1 before GnRHa**	**Time 2 after 8 mo GnRHa**	**Time 3 after 17 mo GnRHa**	**Time 4 after 20 mo GnRHa**	**Threshold value**
Mean fundamental frequency (Hz)	218.682	192.181	187.643	222.98	Average in Brazil: 150–250 Hz for women; 80–150 Hz for men; above 250 Hz for children.
Maximum fundamental frequency (Hz)	229.100	217.98	278.979	242.234	–
Minimum fundamental frequency (Hz)	207.321	174.799	91.64	214.564	–
Jitter percentage (Jitt) (%)	1.196	0.943	1.341	0.729	1.040^*^
Average relative frequency of disturbance (RFD) (%)	0.718	0.488	0.781	0.435	0.680^*^
Pitch perturbation quotient (PPQ) (%)	0.725	0.682	0.868	0.438	0.840^*^
Shimmer (dB)	0.343	0.356	0.528	0.232	0.350^*^

* *Threshold Value: mean values offered by the acoustic analysis program*.

## Background

### Hypothesis of puberty suppression impact in brain white matter

Several studies have been performed in recent years regarding brain changes during puberty, especially when evaluating sex differences (Menzies et al., 2015; Pangelinan et al., 2016; Seunarine et al., 2016; Juraska and Willing, 2017). The DTI approaches are a useful tool for evaluating WM microstructure regarding brain maturation. Sex steroids have been observed to impact axons myelinization and WM microstructure (Mishra et al., 2013; Pesaresi et al., 2015; Pangelinan et al., 2016), in part due to their effects on axonal protein synthesis (Pesaresi et al., 2015). Previous studies have found a positive correlation between age and FA in boys during puberty (Wang et al., 2012), which can be related to the effects of testosterone in axons (Lebel et al., 2008). Other DTI measures, such as the mean diffusivity (MD) and magnetic transfer ratio (MTR) are similarly related to WM maturation (Menzies et al., 2015; Pangelinan et al., 2016) regarding pubertal development. In addition, MD and FA show a trend to be inversely correlated (Clayden et al., 2012; Seunarine et al., 2016). Taken together, these findings suggest a role between puberty and brain maturation, and WM maturation related to androgen exposure during puberty may, at least in part, be accessed by FA. Although there is not a consensus about FA, several studies used it as an experimental measure (Lebel et al., 2008; Clayden et al., 2012; Wang et al., 2012; Menzies et al., 2015; Seunarine et al., 2016).

### Cognitive skills from childhood to adolescence under normal circumstances

The Wechsler tests are among the most widely used instruments for ascertaining intelligence in different populations (Mishra et al., 2013). Evidence for Wechsler Scale validation, considering age as a criterion, is grounded on a theoretic and practical presuppose that intelligence grows between the ages of 8 and 16. The validation for the Brazilian population demonstrated that most of the WISC-IV subtests had a positive and significant correlation with age, indicating that there is a trend for IQ augmentation (Rueda et al., 2013) with aging. The IQ temporal stability between the test and re-test is more reliable according to the individual's age at the first testing (Schuerger and Witt, 1989).

### Review of similar cases: pubertal suppression

Staphorsius et al. (2015) conducted a study in a GD adolescent group under hormonal suppression to investigate the impact of pubertal suppression on executive function (EF). They compared GD adolescents under GnRHa treatment to GD adolescents undergoing physiological puberty and compared them to male and female control groups. They used the Tower of London test and found a negative impact of pubertal suppression on EF. However, they also associate this outcome with a lower IQ before GnRHa treatment.

Recently, studies have shown additional data regarding the impact of steroid deprivation during puberty (Costa et al., 2015; Wojniusz et al., 2016; Hough et al., 2019). In an animal study with pre-pubertal castrated sheep (Hough et al., 2019), researchers reported an impairment in long-term spatial memory that was not reversed by subsequent hormone replacement treatment. Additionally, a global IQ decrease (WISC-III) was reported in a longitudinal follow-up of girls with central precocious puberty (Schuerger and Witt, 1989) treated with GnRHa. Finally, a third study correlated verbal skill impairment to pubertal suppression in a GD group (Costa et al., 2015).

## Discussion

In the present study, we report the lack of significant variation in brain WM FA during pubertal suppression with GnRHa treatment for 28 months in a GD girl. WM brain maturation in some areas during physiological pubertal progression in boys has been previously reported (Menzies et al., 2015; Pangelinan et al., 2016). However, the effects of blocking puberty on brain development and cognition in GD youths still lack conclusive studies (Vries et al., 2011; Wojniusz et al., 2016). The differences in the cognitive skills of boys, girls and GD individuals were previously partially attributed to sexual steroid arousal (Soleman et al., 2013). To our knowledge, this is the first case reporting brain WM and WISC-IV variations during pubertal suppression in a GD youth.

The white matter's FA augmentation during adolescence was previously related to the pubertal stage in male groups (Lebel et al., 2008). Furthermore, Herting et al. (2017) have recently reported a robust statistical model investigating the age-by-sex interaction and gonadal correlation with Fractional Anisotropy, and FA seems to be associated with pubertal development (Herting et al., 2017). The above-mentioned ROIs were chose considering prior publications. The splenium and genu of the CC show an earlier and more rapid increase in FA approximately 11 years old, while variations in FA in other areas are slower, such as for the uncinate and cingulate (Lebel et al., 2008). Other DTI studies also identified continuous WM microstructural developmental changes during adolescence to adulthood (Giorgio et al., 2010). The Tanner stage is an accurate parameter for pubertal evaluation. Prior studies associated this scale to CC's structure and morphology (Chavarria et al., 2014). The results presented here show no increase in the WM FA in a GD girl with suppressed serum testosterone on brain plasticity during male puberty.

The patient's GIQ (global IQ) was further slightly reduced during the follow-up with GnRHa treatment. In fact, the low average GIQ together with impairment in the perceptual organization of intelligence and processing speed index presented even before treatment (T0) suggest that any neurodevelopmental immaturity may have been potentiated by pubertal suppression. Some changes at the functional levels in IQ (e.g., operational memory and EF) can be generally explained by the psychosocial environment or psychopathological status (Cunha, 2000; Roughan and Hadwin, 2011; Zhang et al., 2013; Cromheeke et al., 2014; Li et al., 2016). However, the GD girl did not fully meet any criteria for psychiatric comorbidity during the evaluations. Furthermore, she has shown an improvement in her affective and social life due to the prevention of sexual secondary characteristics arousal.

Some questions emerge from these findings, especially regarding the influence of sex steroids on cognition during puberty. It is likely that the structural and microstructural changes in the brain during adolescence, as discussed above, may interfere on the achievement of complete cognitive potential. Indeed, IQ was recently associated with inter-hemispheric and intra-hemispheric connectivity. Children with high IQ were also those who presented higher FA in some bundles, such as the CC genu and splenium (Nusbaum et al., 2017). These findings highlight the importance of gonadal steroids in brain structure and cognition, and seems to be in accordance with prior study (Seunarine et al., 2016). Neuronal plasticity conferred from sex steroids during puberty may be critical, especially during this period.

Also, it is well known that the brain has different androgen receptor (AR) density, or even lack of AR, along specific areas of white matter and gray matter (Swinft-Gallant and Monks, 2017; Wong et al., 2017). In addition, there might be a synchronism between gray matter and WM development during adolescence (Moura et al., 2017), and these substances might response intrinsically to sex steroids during physiological puberty. In this sense, a plausible explanation for the GIQ decrease should consider a disruption of the synchronic development of brain areas by pubertal suppression. Nevertheless, this is only a speculative discussion about cognition and testosterone. Cognition is more than WISC-IV subtests, and at the present the mechanism for the GIQ decrease observed in this case remains uncertain.

Finally, the patient described here presented a decrease of approximately 30 Hz in the fundamental frequency of the voice during the first year of GnRHa treatment, remaining in the female range. Sex hormones have a substantial influence on voice quality, and testosterone may induce chances in vocal folds, which are parallel to voice pattern changes for fundamental frequencies. At puberty, boys' vocal fold grows up to 1 cm, leading to an average lowering of the fundamental frequency by one octave. In girls, the vocal fold grows less than 4 mm. In this case, the fundamental frequency variation occurred mainly in the first year, and the mean fundamental frequency maintained in the female range during the pubertal suppression. Thus, the testosterone influences over the fundamental frequency results in changes in the voice tone (Nygren et al., 2013; Hari Kumar et al., 2016).

One limitation of the present study is the lack of a paired control without hormone intervention. However, this case report points out the timing interactions between brain maturity, as assessed by FA, cognition and pubertal suppression, and provides us some clues that brain maturation depends also on sex steroids.

## Conclusion

Brain white matter fractional anisotropy remained unchanged in a GD girl during pubertal suppression with GnRHa treatment for 28 months, which may be related to reduced serum testosterone levels. The global performance in the Weschler scale was slightly lower during pubertal suppression compared with baseline, predominantly due to the reduction in operational memory. Either a baseline of a low average cognition or the hormonal status could play a role in cognitive performance during pubertal suppression. The variation in voice frequency was consistent with the testosterone levels and peripheral testosterone effects, as seen in vocal folds. Further longitudinal clinical studies comparing DTI parameters and cognition among TG adolescents under puberty suppression and age-matched controls with physiological pubertal development are needed in order to confirm the present findings and support the hypothesis on the impact of sex hormones on cognition and brain maturity during developmental stages.

## Author contributions

MS: data collection, MRI acquisitions, clinical interview, and literature review. BS: psychologist, WISC-IV evaluation, HTP and Parental stile evaluation. PS: endocrinological follow up, pubertal suppression follow-up. AF: logistics concerns and literature review. MC: DTI analysis and technical support. FT-M DTI analysis and technical support, supervised methodological issues about neuroimage. AC: Ethics and familiar follow up. DdS: literature review, writing manuscript. KS: voice follow up, MRI acquisitions. MA: MRI acquisitions, quality control of MRIs. ST: Childhood psychiatrist evaluated the child during all follow up. ML: chief of the Gender Identity program, family follow up, clinical interviews, literature review, writing the case report. MS, PS, KS, and BS: writing the manuscript. All authors had access to the data and had a role in writing the manuscript. All the authors read and approved the final manuscript.

### Conflict of interest statement

The authors declare that the research was conducted in the absence of any commercial or financial relationships that could be construed as a potential conflict of interest.
